# Mobile-Bearing Total Ankle Replacement In Vivo Kinematic Assessment: A Prospective Study Protocol

**DOI:** 10.3390/jcm11185328

**Published:** 2022-09-10

**Authors:** Silvio Caravelli, Laura Bragonzoni, Emanuele Vocale, Raffaele Zinno, Stefano Di Paolo, Giuseppe Barone, Paolo De Blasiis, Maria Grazia Benedetti, Stefano Zaffagnini, Massimiliano Mosca

**Affiliations:** 1 II Clinic of Orthopaedics and Traumatology, IRCCS Istituto Ortopedico Rizzoli, 40136 Bologna, Italy; 2 Dipartimento di Scienze per la Qualità della Vita, Università di Bologna, 40126 Bologna, Italy; 3 Department of Mental and Physical Health and Preventive Medicine, Section of Human Anatomy, University of Campania “Luigi Vanvitelli”, 81100 Naples, Italy; 4 Physical Medicine and Rehabilitation Unit, IRCCS Istituto Ortopedico Rizzoli, 40136 Bologna, Italy

**Keywords:** radiostereometric analysis, RSA, total ankle replacement, gait analysis, proprioception, clinical outcomes

## Abstract

Total ankle prosthesis as a surgical solution in the case of end-stage osteoarthritis has seen a considerable increase in the last two decades. This study protocol arises from the need to understand the in vivo kinematics of mobile-bearing, flat tibial component total ankle replacement, evaluating the real range of motion and the reciprocal relationships between the components during normal motor tasks through the use of model-based radio-stereometric analysis (MB-RSA). In addition, pre- and post-operative evaluation of walking kinematics with inertial motion sensors, proprioception through a dedicated workstation, and clinical outcomes are discussed. We expect that based on our study protocol researchers will be able to improve future prosthetic designs and validate the setup of MB-RSA, as well as to understand “how an ankle prosthesis moves” once implanted in the patient.

## 1. Introduction

Approximately 15% of the world’s adult population suffer from joint pain and disability caused by osteoarthritis (OA) [1]. Osteoarthritis is the most common form of arthropathy and can affect any joint in the body. Up to 13% of joint localizations affect the ankle [2]. A study by Glazebrook et al. [3] has shown that the health of patients and their quality of life are negatively affected in equal measure by the arthritic degeneration of the ankle and the hip.

Total ankle replacement (TAR) is among the most common elective surgical procedure, aiming at reducing the perceived pain and restoring ankle mobility and function in case of osteoarthritic ankle joints. Over the years, the field of kinematics in total ankle replacement has undergone many changes, and thanks to the initial pioneering experiences, critical issues have been highlighted that have led to the improvement of prosthetic designs and surgical instruments. Although some concepts have now been universally accepted, we are still far from the definition of unique guidelines or the discovery of some prosthetic design that solves all the existing problems.

A considerable effort in this regard was made with the implementation of the dynamic model-based RSA technique, which allows to study in vivo, under weight-bearing and with active muscle action, the biomechanical behavior of the prosthetic components. In this way, it is possible to quantitatively evaluate the data, in terms of 3D rotations and translations of the prosthesis between them.

This paper aimed at presenting a multi-disciplinary protocol to perform a complete clinical and kinematic evaluation of the ankle prosthetic implants, in vivo and in conditions of physiological load. The novelty of this study, compared to previous studies concerning RSA evaluations of TAR (as cited in the Discussion Section), is that it is performed on the most widespread type of prosthesis (three components, mobile-bearing, anterior approach, not anatomical with flat tibia). In addition, it does not aim to evaluate early loosening or micromovements of the bone–prosthesis interface, but the relationships of movement between the individual prosthetic components in vivo, describing the joint kinematics with accuracy.

## 2. Materials and Methods

Ethical approval was sought and granted from the local research Ethics Committee in May 2020 (AVEC CE, Prot. No. 0004698, 139/2020/Sper/IOR). Informed consent will be obtained from all patients before the surgery is scheduled, following the principles of the Declaration of Helsinki. All study data will be treated with maximum confidentiality.

### 2.1. Study Design

This study is a single-center, prospective study. Before surgery and 10 months post-operatively, participants will be evaluated.

### 2.2. Participant Recruitment

Study participation will be proposed to all patients affected by end-stage ankle osteoarthritis with surgical indication by medical staff at the IRCCS Istituto Ortopedico Rizzoli of Bologna after verifying the presence of the inclusion/exclusion criteria (Table 1).

This study is performed by prospectively collecting pre-operative and post-operative data for all patients enrolled. Patients will be enrolled after signing the informed consent form.

### 2.3. Inclusion and Exclusion Criteria

Assessment of the eligibility of each individual patient is based on exclusion and inclusion criteria (non-probability convenience sampling) [4]. Inclusion and exclusion criteria (Table 1) have been identified during preliminary medical assessment. To recruited patients, an identification study code (based on the order of inclusion in the study) has been assigned, and they have been identified only through the individual study code.

Based on the restrictive inclusion criteria and on statistical power analysis, 25 patients will be included in the trial.

### 2.4. Data Collection and Measures

Instruments to record primary and secondary outcome measures and the collection time are summarized in Table 2.

**Figure 1:**
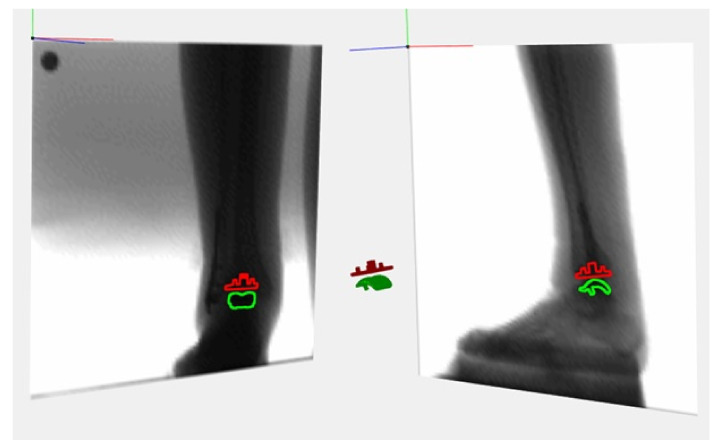
Example of MBRSA 3D reconstruction process of a total joint replacement.

The primary outcome of the study is to quantify the real degree of movements and the overall range of motion of the prosthetic components in vivo using dynamic model-based radio-stereometric analysis (MBRSA). Model-based RSA is gaining popularity as a method that avoids the need for the implantation of tantalum markers, deriving the position of an implant through its X-ray contour. This method is based on minimizing the difference between the virtual projection of a 3D surface of an implant (obtained from computer-assisted design (CAD) models) and the actual X-ray projection (Figure 1).

To this, the possibility of accurately evaluating the kinematic relationships between the components themselves and in the three planes and the antero-posterior/latero-lateral translation degrees of these components is added. Roentgen stereophotogrammetric analysis (RSA) represents a radiological technique, highly accurate to assess the primary stability of the implant (then the micromovements between bone and the prosthetic implant) both for the evaluation of the in vivo kinematics of the prosthetic components [5,6,7,8].

The RSA system involves the simultaneous use of two radiogenic tubes. For the biplanar system, the tubes are oriented perpendicular or 45 degrees to each other. To define the coordinate system of the laboratory, with respect to which the position of the two foci and the X-rays’ geometry is reconstructed, a Plexiglas calibration cage is used during the examination, with a polyhedral shape and with tantalum markers inserted in the thickness of its walls. The markers of the cage that are in the wall adjacent to the X-ray detector are defined fiducial marks, while those on the wall closest to the fires are control points. It is essential that the two X-rays are acquired simultaneously because even a small ankle movement between the two exposures would compromise the accuracy of the results.

**Figure 2:**
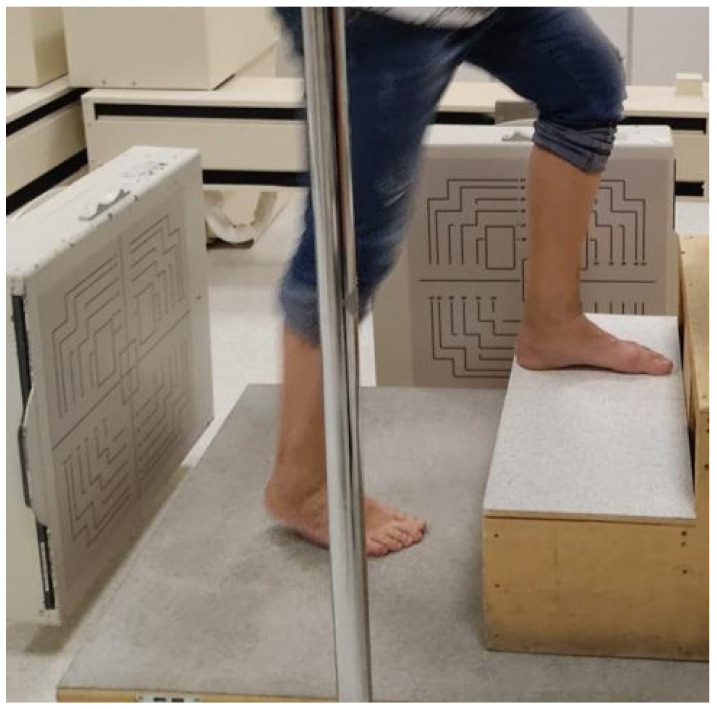
Radio-stereometric fluoroscopy, showing orthogonally positioned X-ray detectors while climbing a step.

The RSA technique in dynamics exploits the same principle of operation as the static system but, unlike the latter, can obtain, in a patient in motion, a series of radiographic frames in sequence (acquisition speed set at 8 fps) in two projections, which are captured simultaneously, orthogonally, and in a single detection (Figure 2).

Each pair of orthogonal projections corresponds to a specific moment of the movement carried out by the patient and will be used to obtain, by means of dedicated software, an instantaneous three-dimensional reconstruction, defined as a “RSA scene”, similar to that described for static RSA and processed in a similar way. The different RSA scenes are then mounted by the same software according to their order of acquisition, obtaining a three-dimensional reconstruction in motion (therefore, dynamic) of the patient’s ankle implants. The 3D reconstructions of metal components are obtained through the use of CAD models. Through this process, position, orientation, and reciprocal movements are obtained between the two joint components. Due to its radiotransparent nature, the position and movement of the polyethylene insert will be indirectly calculated, giving for truth its congruence and stability towards the talar anatomical component and its freedom of sliding towards the flat tibial component.

The motor tasks evaluated during the MBRSA assessment have been identified based on the study biomechanical objectives, the clinical and surgical experience of the researchers, and the typical functional demands of patients undergoing total ankle replacement. These are “down/upstairs” (the patient must descend and/or climb a 16 cm-high step), “overall ROM with open kinetic chain” and “overall ROM with closed kinetic chain”, both in double stance and in single stance. Eventual updates of motor tasks will be considered based on the new literature available and on patients’ physical limitations.

Secondary outcomes, which were recognized to influence quality of life (QoL), will be measured.

The secondary outcomes are represented by the American Orthopedic Foot and Ankle Society (AOFAS) score—ankle hindfoot [9], Short Form-36 (SF-36) [10], the Y-Balance Test (YBT) [11], Delos Postural Proprioceptive System (DPPS) [12,13], and inertial motion sensors.

The AOFAS represents a clinical evaluation system frequently used to evaluate the ankle and hindfoot in an overall way, combining subjective scores related to pain and functional deficits provided by the patient, associated with objective scores obtained by the physical examination performed by the medical doctor. It consists of three subscales for a maximum of 100 points: pain, alignment, and function.

The SF-36 is a self-administered psychometric questionnaire. SF-36 is one of the most widely used questionnaires to measure the health-related QoL. It measures eight scales: mental health (MH), role emotional (RE), social functioning (SF), vitality (VT), physical functioning (PF), general health (GH), bodily pain (BP), and role physical (RP). Component analyses showed that there are two distinct concepts measured by the SF-36: a physical field, represented by the Physical Component Summary (PCS), and a mental field, represented by the Mental Component Summary (MCS) [14].

**Figure 3:**
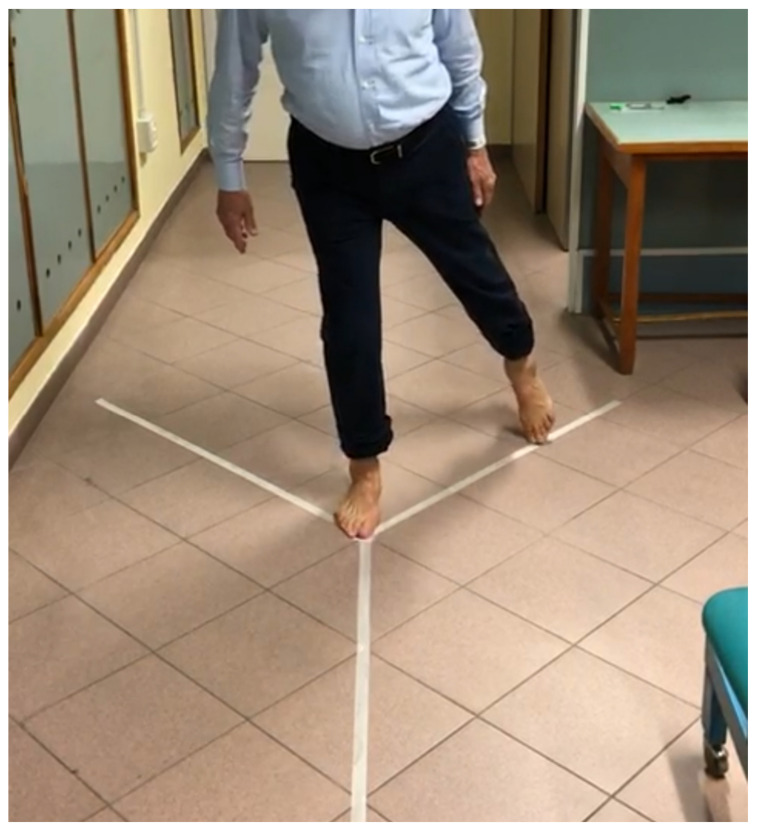
Y-Balance Test.

The Y-Balance Test (YBT) [11] is one of the most widely used tools in the literature to clinically quantify the dynamic balance of the lower limbs, to highlight changes in performance following rehabilitation plans of an injured or operated limb, and to identify what could be an increased risk of injury. The Y-Balance Test is designed to ensure repeatability and uniformity of test administration. It is distinguished from the Star Excursion Balance Test (SEBT) by the presence of three directions that the patient must reach (anterior, posteromedial, posterolateral) (Figure 3). In any case, the two tests are not comparable. This test will be administered for both limbs, healthy and operated.

**Figure 4:**
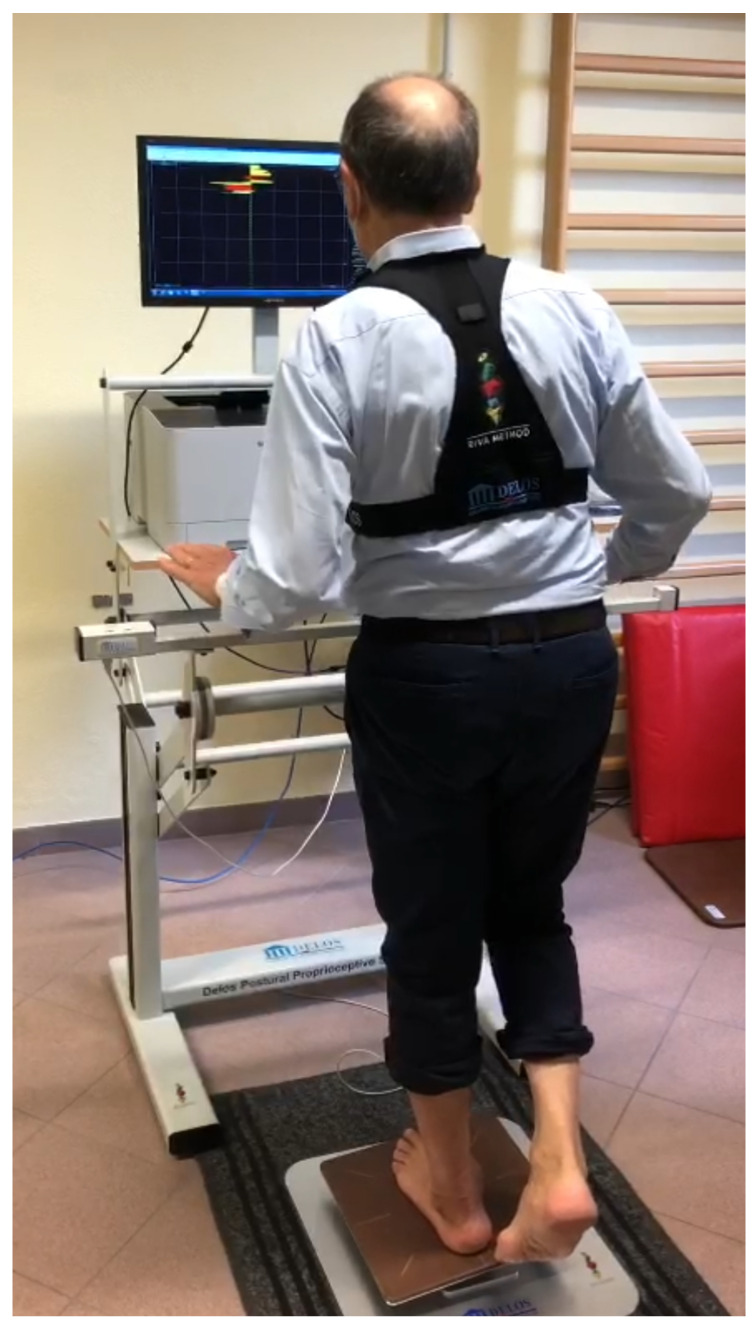
Dedicated Delos workstation.

Proprioceptive stability tests will be performed by DPPS—Delos Postural Proprioceptive System (Delos S.R.L., Turin, Italy). The station is managed by a computer with a specific software (DPPS 6.0, latest version) and includes a Delos Vertical Controller (postural electronic reader (DVC)), an electronic rocking board (unstable proprioceptive board), a flat table, and a monitor. A horizontal bar, the Delos Assistant Desk (DAD), fitted with an infrared sensor, measures how many times and how long a patient leans their hands for support (Figure 4). The DPPS can provide an evaluation of static and dynamic balance, in single- and double-stance positions, and in open- and closed-eyes conditions. The standing balance has been assessed by the validated Stability Index [15].

**Figure 5:**
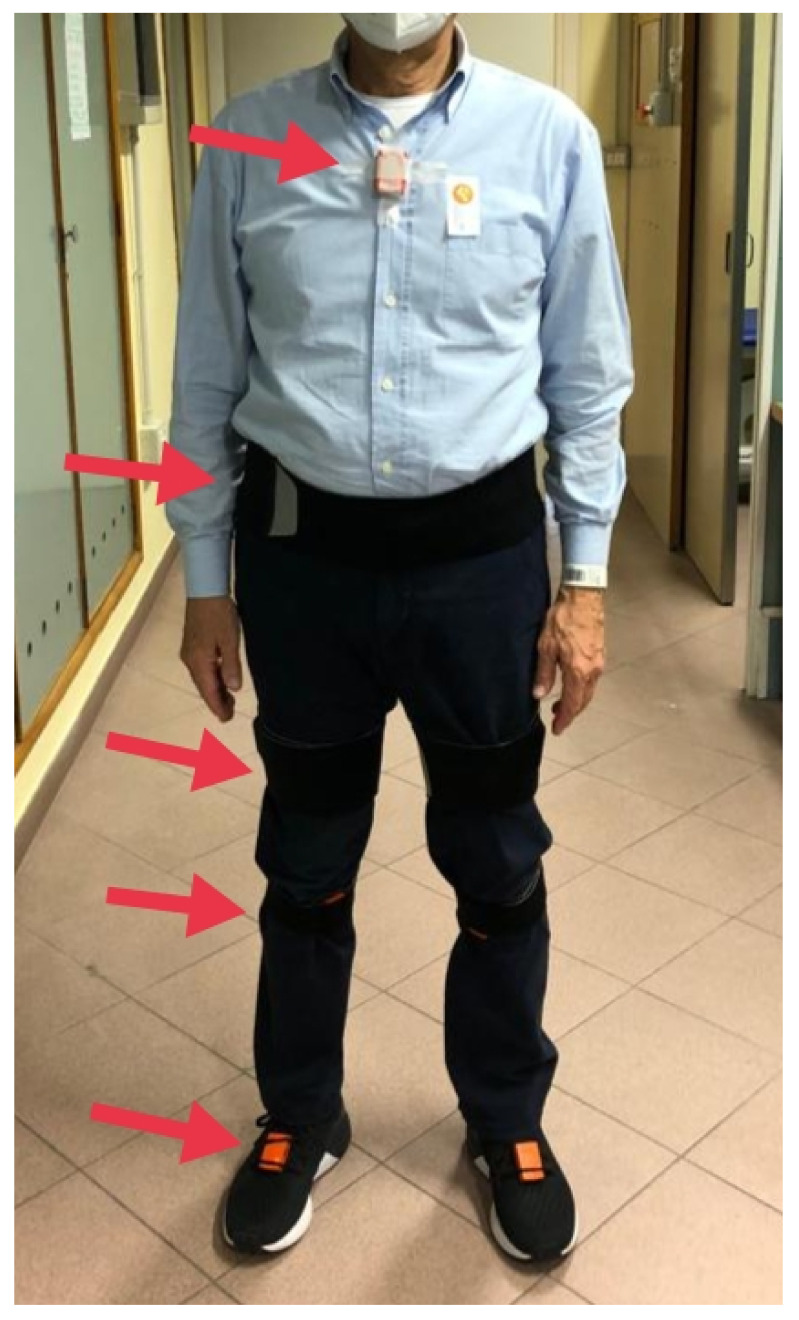
Positioning of the inertial motion sensors.

Full-body gait analysis will be performed through wearable inertial sensors (Xsens Technologies, Enschede, The Netherlands). A set of triaxial wearable inertial sensor units including an accelerometer, a gyroscope, and a magnetometer will be placed on the feet, lower limbs, and trunk (L5 and thorax) (Figure 5). The present system has been validated for this specific task both in healthy and different pathological conditions and will be used to test the patients outside a motion-capture laboratory and guarantee them moving in the most natural way. The gait analysis will be performed before and after the surgical procedure in the hospital hall and will be repeated at a self-selected speed and at the maximum speed possible.

## 3. Discussion

This study protocol presents a trial aimed at evaluating the in vivo clinical and functional status after total ankle replacement. The ankle arthrodesis or fusion has represented, in the past and still now in determined cases, the surgical gold standard for the ankle end-stage osteoarthritis [15,16]. After an ankle arthrodesis, even at 20 years of follow-up, about 90% of satisfaction was reported, but at the same time some studies have shown that in the years following the surgery, the patient may develop a degenerative arthropathy of the adjacent homolateral joints [17,18,19,20]. In recent years, the total ankle replacement has gained considerable popularity but, unlike the knee and hip prosthesis, it is relatively “young”. The total ankle replacement, understood as “resurfacing” of both tibial and talar articular surfaces, was not described until 1973 [21,22]. In the 2000s, a new conception of ankle prostheses (third generation) was born, respecting the normal ligamentous isometry, saving the bone stock and stability of the components [23]. To date, although these new prosthetic designs have shown significant clinical and functional improvements in the patient’s post-operative life, further studies are needed to fill gaps in the understanding of kinematics and in vivo mobility. The same surgical accesses used (anterior or lateral) can probably affect the behavior of the ankle prosthesis during loading and movements of daily life. In this view, a more complete biomechanical evaluation of in vivo ankle prostheses is essential today. An accurate evaluation of the joint replacement kinematics must necessarily consider the movement in its complexity, and then evaluate it qualitatively and quantitatively.

This study aims to assess the complexity of the clinical and kinematic aspects of the total ankle replacement, including the in vivo articular relationships of the implanted components. This will be achieved through the advanced and technologically well-performing tools, as previously listed.

The RSA technique, developed by Selvik in 1974 [24], bases its roots on the mathematical principles of Euler’s classical kinematics, according to which three points not aligned in space uniquely define the position of a rigid body. Using reference points, it is possible to measure the macro- and micro-movements that take place between several contiguous elements. The RSA technique has allowed the acquisition of important information in different fields of application [25,26,27,28,29,30,31], with results of higher accuracy than those obtained by traditional radiography or fluoroscopic, with lower costs compared to dynamic MRI [32,33] and without the limitations of the latter technique related to the impossibility of studying patients already treated with metal devices, such as prostheses and hardware. A study related to in vivo kinematic assessment of ankle prosthesis was published in 2012 by Cenni et al. Although using a similar technique, the authors examined 12 patients undergoing TAR surgery with a ligament-compatible three-component prosthetic design exclusively using video-fluoroscopy. The study revealed the range of translation and rotation of the prosthetic components on the three planes similar to the physiological movement. The data reported at a follow-up of 12 and 24 months indicated satisfactory clinical and kinematic results [34]. Compared to the study of Cenni et al., the protocol described in this paper shows the different methods of clinical, functional, and kinematic investigation aimed at the evaluation of multiple and diversified aspects.

Additional methods and tests are added to this method to evaluate the biomechanics and stability of the substituted ankle.

The YBT has been shown in the literature to have excellent intra- and inter-operator reproduction. In addition, it is easy to run by healthcare personnel to assess the dynamic balance, essential for making clinical and surgical decisions.

In this study, the possibility of evaluating postural control through the result of visual, vestibular, and proprioceptive affections of the patient has been ensured by the use of the DPPS. This electronic system allows to calculate the risk of fall and injury based on the preventive behavioral strategies of the patient. In previous studies, the Delos system has been used to evaluate the effect of visuo-prospective training on the risk of falls [35] as well as to train proprioception in team-sport players. Since DPPS also allows to evaluate single-stance stability, it is of crucial importance for risk assessment and rehabilitation strategies of ankle sprain or other musculoskeletal-related injuries in sports [36,37]. For the purpose of this protocol, the DPPS outcomes will be analyzed to compare the prosthetic and healthy ankle, and pre- and post-surgery.

The gait analysis is a common quantitative measurement applied to a wide variety of different musculoskeletal and neurological pathologies. It allows to investigate a simple task such as the gait and detect asymmetries and postural compensations. The use of wearable sensors will allow to test the patients in a familiar environment, such as the hospital hall, instead of a specialized motion-capture laboratory. Such an analysis will allow investigating the kinematical differences between the two limbs at a single time and before and after the surgical procedure. These differences will not be investigated only at the ankle level, but will allow determining possible compensations with the knee, hip, and trunk caused by the pathology and/or the surgery. The investigation of two different walking speeds (self-selected and maximum possible) will provide further insights on the confidence patients have on their pathological ankle and will resemble multiple different daily life conditions.

This study has some limitations. MBRSA has been shown to be effective and sufficiently accurate [38,39] in the quantification of the reciprocal translation and movement between the components, as is the objective of this study, but it subjects patients to radiological exposure, it has a limited field of view, and software reconstructions require large amounts of working hours, trained personnel, and hardware to develop.

A further limitation of this trial is that the post-operative rehabilitation protocol followed by individual patients may not be standardized. Each of them has carried out a rehabilitation plan at home, in accordance with the postoperative instructions issued by the medical staff.

This may represent a bias in the assessment of kinematic outcomes at the end of the follow-up.

Several other radiological methods have been used for the evaluation of the prosthetic joint components, in particular regarding micro-movements at the bone–prosthesis interface and regarding the wear, or other orthopedic surgical procedures. Among the most described, we find CT-based analysis [40,41,42]. Despite the described value of this method, a higher radiant dose was reported in the literature than in RSA [41]. In addition, to our knowledge, other 3D radiological analysis techniques aimed at the exclusive study of prosthetic joint kinematics have not been described.

The future results of this study protocol are expected to show the importance to monitor the joint function after total ankle replacement with different quantitative assessments.

The use of inertial sensors to evaluate the gait before and after surgery is expected to provide quantitative and solid information on kinematic patterns after TAR. Post-surgery, the RSA should allow a quantitative evaluation of the ankle kinematics during a motor task that is fundamental for monitoring the implant conditions.

## Figures and Tables

**Table 1:** Inclusion and exclusion criteria.

Inclusion Criteria	Exclusion Criteria
· Signed informed consent · End-stage ankle osteoarthritis · Primary total ankle replacement · Age between 40 and 80 years · BMI < 40 kg/m^2^ · Patients physically and mentally inclined and able to participate in post-operative rehabilitation and clinical follow-up	· Severe ankle malalignment (>10°) · Neuromuscular diseases · End-stage knee or hip osteoarthritis (Kellgren–Lawrence > 3) · Talar avascular necrosis or other ankle bone loss · Local or systemic infections · Alcoholism, drug or intravenous substance abuse, psychosis, personality disorder/s · Participation in any other trial of another drug or device experimentation within 60 days prior to the screening visit · Clinically documented acute or chronic pathology which may affect life expectancy or make interpretation of the results difficult · Pregnancy confirmed by positive serum Beta-Hcg or ongoing breastfeeding · Severe ankle instability

**Table 2:** Outcome timeline.

Outcome Assessment	Baseline (T0)	10 Months (T1)
MB Radio-stereometric Analysis		X
Delos Balance Assessment	X	X
Y-Balance Test	X	X
AOFAS score	X	X
SF-36	X	X

## Data Availability

Not applicable.
